# Computational prediction of CNP0387675 as a non-nucleoside inhibitor of MraY, from natural product-based multi-template screening against *Pseudomonas aeruginosa*

**DOI:** 10.3389/fmicb.2025.1648850

**Published:** 2025-09-05

**Authors:** Zeng Qingxin, Dong Li, Aotian Guo, Haichuan Hu, Zhengwei Huang, Tao Shen

**Affiliations:** ^1^Department of Thoracic Surgery, Sir Run Run Shaw Hospital, Zhejiang University School of Medicine, Hangzhou, China; ^2^Functional Inspection Department, Hangzhou Linping Hospital of Traditional Chinese Medicine, Hangzhou, China

**Keywords:** MraY, *Pseudomonas aeruginosa*, antimicrobial resistance, pharmacophore screening, multi-template docking, molecular dynamics simulations, drug-likeness

## Abstract

Antimicrobial resistance driven by multidrug-resistant Gram-negative bacteria, notably *Pseudomonas aeruginosa*, urgently necessitates novel antibacterial targets and inhibitors. MraY, an integral membrane enzyme catalyzing lipid I formation in peptidoglycan synthesis, represents an attractive antibacterial target. In the absence of experimentally resolved structures for *P. aeruginosa* MraY, we developed a computational pipeline integrating multi-template homology modeling, pharmacophore-guided virtual screening, multi-template docking, molecular dynamics (MD) simulations, and medicinal chemistry profiling to identify structurally novel inhibitors. The compound CNP0387675, identified through pharmacophore-driven multi-template docking, exhibited stable binding interactions with conserved catalytic residues (ASP-195, ASP-267), validated through extensive MD simulations. Remarkably, CNP0387675 represents a non-nucleoside inhibitor, structurally distinct from traditional nucleoside-based inhibitors, thereby circumventing typical drug-likeness limitations and potential off-target toxicities associated with nucleoside analogs. Our findings underscore the potential of computationally guided, structure-based discovery strategies for novel antimicrobial scaffolds, providing critical insights and candidate inhibitors suitable for subsequent experimental validation against resistant Gram-negative pathogens.

## 1 Introduction

The emergence and global spread of antimicrobial resistance pose a severe threat to public health and have significantly diminished the efficacy of conventional antibiotics (Ahmed et al., 2024). The rapid evolution of resistance mechanisms—especially among Gram-negative pathogens such as *Pseudomonas aeruginosa*—has outpaced the discovery of novel antibacterial agents (Wang et al., 2024). These pathogens often exhibit multidrug resistance through a combination of efflux pumps, porin mutations, and antibiotic-modifying enzymes, making the development of antibiotics with new modes of action an urgent priority (Macesic et al., 2025; Gaurav et al., 2023).

MraY (phospho-N-acetylmuramoyl-pentapeptide-transferase) has recently gained traction as a promising antibacterial target (Yamamoto et al., 2024). This integral membrane enzyme catalyzes the first committed membrane step in bacterial cell wall biosynthesis: the transfer of phospho-N-acetylmuramoyl-pentapeptide to undecaprenyl phosphate, forming lipid I. MraY is universally conserved among bacteria and is essential for cell viability (Bouhss et al., 2004). Importantly, its catalytic mechanism and structural features are distinct from any human homologs, making it an attractive target for selective antimicrobial intervention (Chung et al., 2016; Mashalidis and Lee, 2020). A number of nucleoside-based natural products—such as muraymycins (McDonald et al., 2002), mureidomycins (Isono et al., 1989), capuramycins (Yamaguchi et al., 1986; Cai et al., 2015), and tunicamycins—have been identified as potent MraY inhibitors, with several displaying promising *in vitro* antibacterial activity against resistant strains (Chung et al., 2016).

Despite the validated relevance of MraY as a drug target, structure-based discovery of new inhibitors remains challenging (Manning et al., 2023). First, MraY’s conformational plasticity complicates the definition of a universal binding pocket, especially in the absence of a bound ligand. Second, while structural information is available for MraY from *Aquifex aeolicus* and several Gram-positive bacteria, no experimental structure exists for MraY from *Pseudomonas aeruginosa*—one of the most clinically significant Gram-negative organisms. Specifically, MraY from *P. aeruginosa* (UniProt ID: Q9HVZ8) lacks crystallographic or cryo-EM characterization, limiting rational drug design efforts targeting this species (Chung et al., 2016).

To address these limitations, we adopted a computational approach to identify natural product-like inhibitors of *P. aeruginosa* MraY. Homology models of Q9HVZ8 were constructed based on eight diverse ligand-bound MraY crystal structures. This multi-template modeling strategy allowed us to capture relevant structural heterogeneity and construct a receptor ensemble for downstream analysis. Pharmacophore hypotheses derived from conserved ligand interactions were used to screen a structurally diverse natural product library, resulting in the identification of CNP0387675 (1-(pyridine-4-carbonylamino)-3-[(2R,3R,4S,5R,6R)-3,4,5-trihydroxy-6-(hydroxymethyl)tetrahydropyran-2-yl]urea) as a top-ranked candidate.

Subsequent multi-template docking, induced fit refinement, and long-timescale molecular dynamics simulations revealed that CNP0387675 not only forms a stable binding conformation but also induces a conformationally restricted, low-energy state of the protein. The ligand’s interactions were dissected through energy decomposition, cross-correlation dynamics, and alanine scanning mutagenesis, confirming the structural indispensability of key residues such as LYS-119, ASP-195, and ASP-267. Binding pose metadynamics and free energy landscape mapping further validated the persistence and robustness of the interaction network.

In summary, this study integrates structure-based modeling, ensemble virtual screening, and dynamic simulation techniques to propose CNP0387675 as a potential *MraY* inhibitor against *P. aeruginosa*. These findings provide a mechanistic framework for the rational design of new antibacterial agents targeting lipid I biosynthesis in resistant Gram-negative pathogens.

## 2 Methods

### 2.1 Homology modeling

The amino acid sequence of *Pseudomonas aeruginosa* MraY (UniProt ID: Q9HVZ8) was retrieved from the UniProt database^1^. Eight crystal structures of MraY orthologs complexed with antibiotics—PDB IDs: 5CKR (Chung et al., 2016), 5JNQ (Hakulinen et al., 2017), 6OYH, 6OYZ, 6OZ6 (Mashalidis et al., 2019), 8CXR (Nakaya et al., 2022), 9B70, and 9B71 (Yamamoto et al., 2024)—were selected as structural templates from the Protein Data Bank^2^. Homology modeling was performed using the Prime module in Schrödinger Suite 2025–1. Each template was aligned with the MraY target sequence, followed by loop refinement and energy minimization using the OPLS4 force field (Lu et al., 2021). Model quality was assessed through the SAVES v6.0 server, including PROCHECK (Hodsdon et al., 1996), VERIFY3D (Lüthy et al., 1992), and ERRAT (Fatima et al., 2024) evaluations. Only high-quality models with favorable stereochemistry and fold integrity were retained for downstream analysis.

### 2.2 Complex refinement

Each homology model was refined using the Refine Protein–Ligand Complex module in Schrödinger 2025–1. Side chains within 5 Å of the bound ligand were flexibly optimized while constraining the protein backbone. The OPLS4 force field and VSGB 2.1 implicit solvation model (Tripathi et al., 2025) were applied. Refined models were selected based on minimized strain and retention of critical ligand–protein interactions. Strain was evaluated based on the total potential energy of the refined complex, with models showing high steric clashes or unusually elevated energy excluded. Key interactions were defined as hydrogen bonds or electrostatic contacts with catalytically conserved residues such as ASP-195, ASP-267, and LYS-119. When multiple models exhibited comparable strain (within ~5 kcal/mol), preference was given to those preserving more critical interactions. Models failing both criteria were discarded.

### 2.3 Pharmacophore model construction

Pharmacophore models were constructed using the Phase module in Schrödinger 2025–1 based on the eight refined MraY–ligand complexes. Interaction features such as hydrogen bond donors/acceptors, aromatic rings, hydrophobic regions, and ionizable groups were extracted and clustered. Up to seven features were retained per complex to maintain model interpretability. A consensus pharmacophore was generated by aligning and merging conserved features across all structures.

For validation, decoy molecules corresponding to the eight co-crystallized ligands were generated using the DUD-E (Database of Useful Decoys: Enhanced) server (Mysinger et al., 2012; Stein et al., 2021). The pharmacophore model’s discriminative power was assessed by enrichment factor and ROC curve analyses comparing active ligands and their decoys.

### 2.4 Pharmacophore-based virtual screening

The consensus pharmacophore model was applied to screen natural products from the COlleCtion of Open Natural prodUcTs (COCONUTS) database (Chandrasekhar et al., 2025). Ligands were prepared using LigPrep, with Epik-generated tautomers and protonation states at pH 7.0 ± 2.0 (Johnston et al., 2023). Phase was used to map pharmacophore features with up to one missing element allowed. Hits were ranked by Phase Screen Score, and compounds matching ≥5 of 7 features were retained for structure-based docking.

### 2.5 Structure-based docking

Docking of pharmacophore-filtered hits was performed using the Glide module in Schrödinger 2025–1 against all eight homology models. Receptor grids were centered on the binding sites of the co-crystallized ligands using the Receptor Grid Generation panel. A 20 Å × 20 Å × 20 Å inner grid box was defined around the centroid of the reference ligand, with an outer grid box extending 10 Å beyond. The van der Waals scaling factor was set to 1.0 with no partial charge cutoff. No constraints were applied to hydrogen bonding or rotatable groups. Water molecules were excluded.

An ensemble docking strategy was used, in which each compound was docked across eight distinct homology models representing different ligand-bound conformations of MraY. Ensemble docking utilizes multiple receptor conformations—either computational or experimental—to better account for structural flexibility and binding-site diversity (Amaro et al., 2018).

Standard precision (SP) docking was used for initial screening. Docking scores of the redocked co-crystallized ligands were used as performance benchmarks, and only compounds scoring equal to or better than these ligands were retained. Final hits were deduplicated and prioritized based on docking pose quality and chemical diversity. Pose quality was assessed by Glide docking score and visual inspection of interactions with key catalytic residues such as ASP-195, ASP-267, and LYS-119. Chemical diversity was evaluated using ECFP4 fingerprints and Tanimoto similarity (threshold = 0.6), followed by Butina clustering. When docking scores were within 1.0 kcal/mol, preference was given to top-scoring compounds with more distinct scaffolds to ensure structural diversity in the final selection.

### 2.6 Induced fit docking and model selection

To capture induced fit effects and further refine complex models, Induced Fit Docking (IFD) was conducted on both co-crystallized ligands and the intersection of pharmacophore- and docking-selected compounds. Side-chain flexibility within 5 Å of the ligand was permitted, followed by Prime refinement and redocking. The best protein–ligand pairs were selected based on Glide scores and binding pose quality. A two-tailed paired t-test was used to evaluate score distributions and identify statistically significant pose improvements (Miller et al., 2021).

### 2.7 Molecular dynamics simulation in a membrane environment

The selected MraY–ligand complexes were subjected to all-atom molecular dynamics simulations using the Desmond module in Schrödinger 2025–1. A pre-equilibrated POPC lipid bilayer was inserted around each membrane-spanning MraY structure using the System Builder tool. The systems were solvated with TIP3P water and neutralized with 0.15 M NaCl. The OPLS4 force field was used for all components. After a 10-stage relaxation protocol, simulations were carried out in the NPT ensemble at 310 K and 1 atm using the Nose–Hoover thermostat and Martyna–Tobias–Klein barostat, following the approach described in Xu et al. (2024).

Trajectory analysis included RMSD, RMSF, ligand RMSD, and protein–ligand contacts. Dynamic cross-correlation matrix (DCCM) analysis was conducted using Bio-3D to identify correlated residue motions (Grant et al., 2006). Free energy landscape (FEL) analysis was performed using PCA-derived principal components to visualize dominant conformational basins and binding-state transitions.

### 2.8 Alanine scanning and binding pose metadynamics

To assess residue-level contributions to binding stability, key interacting residues were subjected to alanine scanning mutagenesis using Maestro. Each mutant was subjected to 10 independent 10 ns binding pose metadynamics (BPMD) simulations in Schrödinger, following initial energy minimization. The RMSD of the ligand from its initial pose was used as the collective variable (CV), and five replicates were conducted for each wild-type and mutant complex. PoseScore (average CV RMSD), PersScore (percentage of frames within 2 Å), and CompScore (composite metric) were calculated. Increased pose instability in mutant systems indicated the importance of the corresponding residue for ligand anchoring (Lukauskis et al., 2022).

### 2.9 *In silico* ADMET and drug-likeness evaluation

Physicochemical properties, drug-likeness parameters, and medicinal chemistry profiles of CNP0387675 and the 9B71 Analogue 3 were predicted using ADMETlab 3.0^3^, an online platform for integrated ADMET property prediction. The platform evaluates a wide range of descriptors, including molecular weight, hydrogen bond donors/acceptors (HBD/HBA), topological polar surface area (TPSA), logP, logS, logD, and pKa values. In addition, drug-likeness filters such as the Lipinski, Pfizer, and GSK rules were assessed, along with synthetic accessibility scores (SAscore, GASA), structural alerts (e.g., PAINS, BMS rule), and assay interference risks (e.g., colloidal aggregation, fluorescence). Quantitative estimates of drug-likeness (QED), natural product-likeness scores (NPscore), and molecular complexity (Fsp^3^, MCE-18) were also computed. Default parameters were used for all calculations.

### 2.10 Bacterial strains and culture

*Pseudomonas aeruginosa* PAO1 lineage strains (ATCC 15692 and its historical aliases 1C and PRS 101) were expanded aerobically at 37 °C on Nutrient broth/agar according to the ATCC recommendations (Medium 3 or Medium 129). These conditions were chosen to maintain continuity with downstream broth microdilution workflows. The strict anaerobe Enterocloster (Clostridium) bolteae 90A9 was cultured at 37 °C under strict anaerobiosis following DSMZ guidance, using Wilkins–Chalgren Anaerobe medium (DSMZ Medium 339) or Fastidious Anaerobe medium (DSMZ Medium 1203) as appropriate. Anaerobe handling was performed under reduced conditions in accordance with DSMZ instructions for cultivation of anaerobes.

### 2.11 Broth microdilution susceptibility testing and OD600 readout

Antimicrobial susceptibility testing followed Clinical and Laboratory Standards Institute (CLSI) reference procedures. Aerobic testing for PAO1 adhered to CLSI M07 using twofold serial dilutions in 96-well format with cation-adjusted Mueller–Hinton broth and a target inoculum of ~5 × 10^5 CFU/mL (acceptable range 2–8 × 10^5 CFU/mL). Anaerobe testing for E. bolteae referenced CLSI M11; cultures were prepared and incubated under strict anaerobiosis with matched inoculum and appropriate broth, and the reference agar-dilution method was considered where indicated by the standard. Plates included a sterile blank and a solvent control (≤1% v/v DMSO). After 18–20 h incubation, the minimum inhibitory concentration (MIC) was defined as the lowest concentration with no visible growth per CLSI criteria, and MIC values were interpreted in the context of CLSI performance standards. For visualization of concentration–response behavior, OD600 was measured at 18 h and normalized to the solvent control to generate dose–response curves; a horizontal line corresponding to 10% of the control mean OD was overlaid as an operational “no-growth” threshold for plotting only. Formal susceptibility reporting relied exclusively on the visible-growth MIC endpoint as specified by CLSI.

## 3 Results

### 3.1 Structural evaluation of homology models of *P. aeruginosa* MraY for structure-based antibiotic discovery

To compensate for the lack of experimentally determined structures of *P. aeruginosa MraY* from strain ATCC 15692 and related isolates, we constructed homology models using eight available crystal structures of *MraY* in complex with various ligands as templates. Although recent advances such as AlphaFold have greatly improved protein structure prediction, we deliberately chose not to employ AlphaFold-predicted structures for *MraY* in this study. AlphaFold models are typically generated in the apo (ligand-free) state and may not accurately capture the biologically relevant conformations associated with ligand binding, especially for virtual screening and docking applications. As an integral membrane protein, *MraY* exhibits pronounced conformational plasticity in its binding regions, and ligand-induced structural adaptations are critical for defining druggable pockets. AlphaFold predictions, while valuable for fold assessment, lack these ligand-induced features and thus may be less reliable for the purposes of structure-based drug discovery targeting *MraY*. Therefore, we selected experimentally determined ligand-bound templates for homology modeling to ensure a more accurate and relevant representation of the binding environment. The corresponding structural evaluation metrics are summarized in Table 1. ERRAT analysis revealed Overall Quality Factors ranging from 89.66 to 97.12 across all models, indicating that backbone environments and non-bonded atomic interactions were geometrically reasonable and devoid of major steric clashes. These values far exceeded the generally accepted reliability threshold of 90, demonstrating excellent stereochemical reliability and backbone geometry across models. Minor fluctuations in ERRAT scores are likely attributed to differences in template resolution and sequence alignment coverage rather than inherent flaws in the modeling process.

**Table 1:** Quality assessment of eight MraY homology models from *P. aeruginosa* using ERRAT and Ramachandran plot analyses.

Template	Sequence identity	ERRAT score	Favored regions	Additional allowed regions	Generously allowed regions	Disallowed regions
5CKR	0.480	94.225	0.916	0.074	0.010	0.000
6OYH	0.480	95.308	0.884	0.097	0.013	0.006
6OZ6	0.480	92.733	0.910	0.074	0.016	0.000
9B71	0.490	90.712	0.913	0.077	0.003	0.006
9B70	0.490	93.636	0.919	0.068	0.013	0.000
8CXR	0.480	97.125	0.88	0.110	0.003	0.007
6OYZ	0.480	94.801	0.906	0.074	0.013	0.006
5JNQ	0.400	89.655	0.858	0.119	0.016	0.006

Ramachandran plot analysis further supported the structural integrity of the models. The percentage of residues occupying the most favored regions consistently exceeded 85%, with the majority of models achieving values above 90%. Meanwhile, residues located in disallowed regions averaged only 0.39%, underscoring a high degree of torsional angle consistency and a lack of local geometrical strain. This is particularly notable given the complexity of accurately modeling integral membrane proteins such as *MraY*, where transmembrane helices and flexible loop regions often pose significant structural challenges.

Taken together, these results confirm that all eight MraY homology models possess robust stereochemical integrity and global structural reliability. Rather than representing conflicting alternatives, the subtle differences among these models reflect complementary conformational hypotheses, each capturing distinct features of the ligand-binding environment. From a computational perspective, employing an ensemble of independently validated single-template models helps mitigate template selection bias and receptor conformational bias, both of which are common limitations in single-structure approaches. This ensemble strategy enhances the robustness of downstream tasks such as pharmacophore-based virtual screening, induced-fit docking, and conformational sampling. Collectively, these models provide a structurally reliable platform for the rational discovery and optimization of novel MraY-targeting inhibitors.

In light of the validated structural reliability of all eight models, we proceeded to conduct large-scale virtual screening of natural product libraries across the entire ensemble to capture diverse yet biologically relevant binding scenarios.

### 3.2 Pharmacophore model evaluation identifies AAAR_3 as an optimal template for *MraY* inhibitor discovery

Building upon validated homology models of *P. aeruginosa* MraY, we next sought to identify essential pharmacophoric features derived from known co-crystallized inhibitors. To achieve this, we generated a series of pharmacophore hypotheses from eight distinct ligand-bound MraY homology models, subsequently evaluating their predictive power using four complementary scoring metrics: BEDROC (early enrichment capability), PhaseHypoScore (model robustness and alignment quality), Site Score (geometric consistency), and Survival Score (applicability in virtual screening) (Table 2).

**Table 2:** Performance evaluation of pharmacophore models derived from eight ligand-bound MraY structures using BEDROC, PhaseHypoScore, Site Score, and Survival Score.

Title	BEDROC score	PhaseHypoScore	Site score	Survival score
AAAR_3	1	1.256	0.975	4.268
AAADR_7	0.95	1.214	0.945	4.395
AADR_3	0.931	1.185	0.986	4.236
AAAR_1	0.919	1.177	0.962	4.298
AAADR_5	0.913	1.177	0.94	4.401
AADR_4	0.912	1.165	0.95	4.226
AAADR_4	0.886	1.15	0.953	4.406
AADDR_3	0.882	1.146	0.966	4.394
AAADR_2	0.878	1.144	0.971	4.425
AAADR_6	0.853	1.117	0.916	4.399
AAADR_1	0.85	1.116	0.937	4.437
AADDR_1	0.824	1.089	0.895	4.424
AAAR_4	0.826	1.081	0.959	4.239
AAADR_3	0.805	1.07	0.983	4.411
AAAR_2	0.77	1.026	0.987	4.278
AADR_1	0.767	1.022	0.978	4.249
ADDR_1	0.733	0.989	0.964	4.275
AADR_5	0.729	0.983	0.944	4.22
AADDR_2	0.635	0.899	0.952	4.397
AADR_2	0.641	0.896	0.978	4.24

Pharmacophore models were constructed based on ligand-bound MraY templates. Each model is labeled using a four-letter code representing its pharmacophore features (e.g., “AAAR” indicates two hydrogen bond acceptors, one projected acceptor, and one aromatic ring). Among all generated hypotheses, AAAR_3 exhibited the best overall performance in terms of early enrichment, geometric consistency with known MraY inhibitors, and screening robustness, and was therefore selected as the representative model for virtual screening.

Pairwise structural alignment of the eight homology models revealed that although the transmembrane backbone architecture was conserved, significant conformational variability was observed in flexible loop regions proximal to the ligand-binding site, notably residues 65–80, 190–200, and 260–270. Additionally, subtle yet important differences in side-chain orientations of catalytic residues (ASP-195, ASP-267, and LYS-119) were evident across models. Rather than reflecting modeling inconsistencies, these structural variations arose naturally from the conformational adaptability driven by the distinct ligand-bound templates. Consequently, these local conformational differences directly influenced the spatial arrangement of pharmacophore features and the overall shape of the binding pocket, resulting in varied pharmacophore hypotheses.

**Figure 1:**
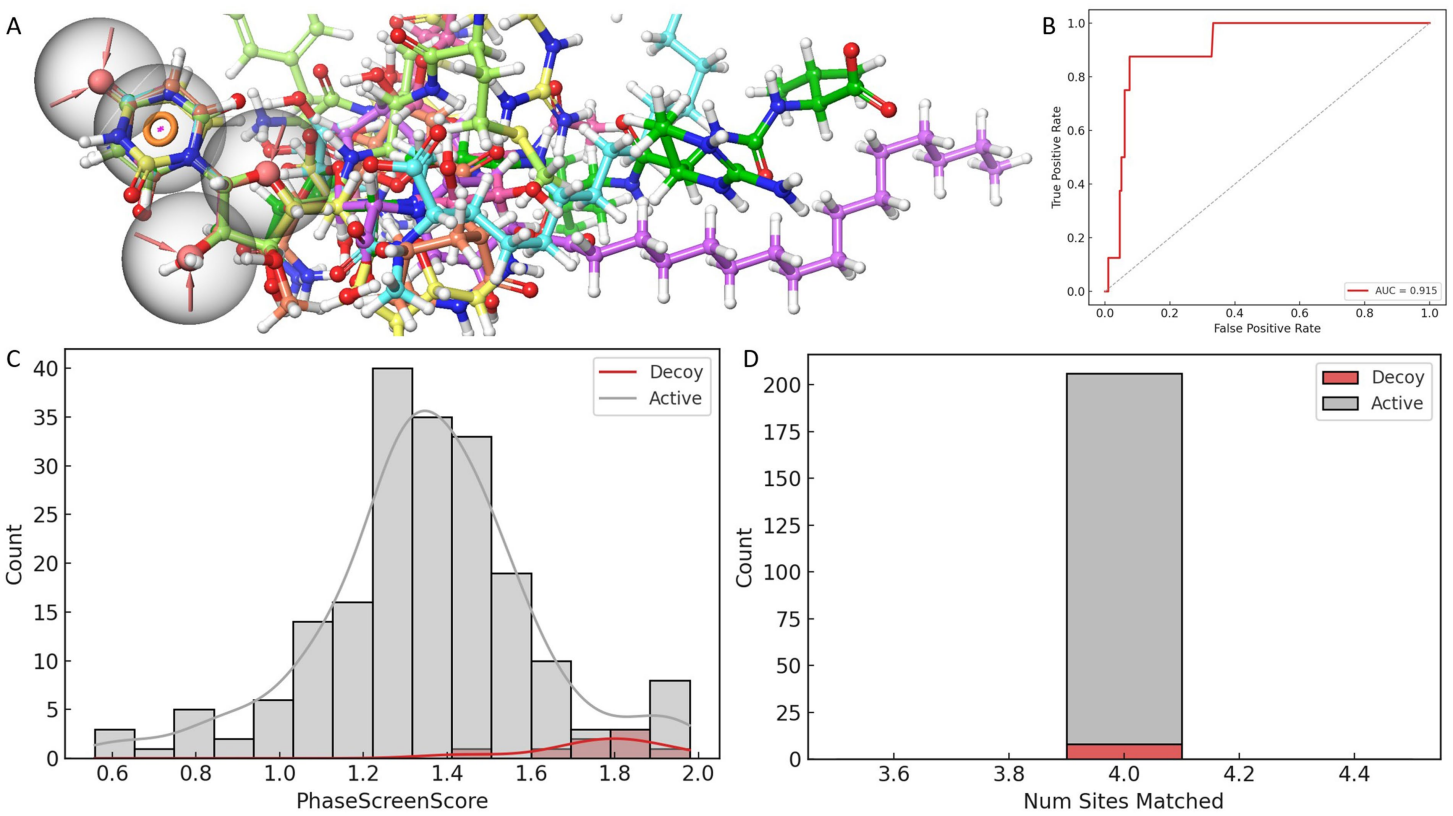
Evaluation and validation of the AAAR_3 pharmacophore model. **(A)** Comparative performance of pharmacophore models constructed from eight ligand-bound *MraY* crystal structures, assessed using four scoring metrics. **(B)** ROC curve analysis of the AAAR_3 model against a dataset of actives and decoys. **(C)** Distribution of PhaseScreenScores for active compounds and decoys (lower scores indicate better fit to the pharmacophore model). **(D)** Comparison of the number of pharmacophore features matched by active compounds versus decoys.

Among all pharmacophore models evaluated, AAAR_3 consistently demonstrated superior performance across multiple metrics. Specifically, it achieved an optimal BEDROC score of 1.000, indicating exceptional early enrichment and prioritization of active compounds, along with the highest PhaseHypoScore (1.256), reflecting excellent coverage and alignment fidelity of pharmacophoric features. Additionally, AAAR_3 obtained a strong Site Score (0.975), suggesting robust geometric consistency of its pharmacophore arrangement, and a high Survival Score (4.268), affirming its practical robustness in screening applications. Although certain models, such as AAADR_1 and AAADR_2, displayed marginally higher Survival Scores, their relatively lower BEDROC and PhaseHypoScores indicated reduced reliability in early-stage compound prioritization. Therefore, AAAR_3 emerged as the most balanced and effective pharmacophore hypothesis, demonstrating optimal predictive power, internal consistency, and structural interpretability (Figure 1A).

Further validation of AAAR_3’s reliability was conducted using a curated dataset comprising known active compounds derived exclusively from nucleoside-based ligands previously reported to inhibit MraY orthologs (including those from *Aquifex aeolicus* and various Gram-positive bacteria), along with carefully generated decoys structurally similar to these nucleoside-based inhibitors. ROC analysis indicated strong discriminatory capability (AUC = 0.928), while the BEDROC score at *α* = 20 (0.025) revealed acceptable early enrichment performance, given the limited size of the validation dataset (Figure 1B). Importantly, statistical analysis (Mann–Whitney U test, *p* = 3.5 × 10^−5^) demonstrated that active compounds achieved significantly higher PhaseScreenScores compared to decoys, underscoring AAAR_3’s effectiveness in discriminating bioactive molecules from chemically similar yet inactive counterparts (Figure 1C). Furthermore, active compounds predominantly matched all four pharmacophore features, whereas decoys exhibited a broader and less specific feature-match distribution (Figure 1D), although without statistical significance (*p* = 1.0). Collectively, these analyses confirmed the robustness and reliability of AAAR_3 for application in structure-based virtual screening.

It is noteworthy that the spatial distribution of AAAR_3 pharmacophore features was intentionally confined primarily to a localized region derived from conserved ligand-core interactions. Specifically, the model emphasized conserved hydrogen-bond acceptors and aromatic ring interactions with catalytically critical residues (ASP-195 and ASP-267). Although AAAR_3 did not fully capture peripheral ligand interactions, this focused design served as a high-specificity initial filter aimed at identifying compounds capable of productive core-site engagement. Comprehensive evaluations of peripheral interactions and binding pocket occupancy were subsequently addressed through induced-fit docking and molecular dynamics simulations, providing a holistic assessment of ligand flexibility and structural compatibility.

In summary, pharmacophore model AAAR_3 emerged as the optimal template for subsequent multi-template virtual screening against a natural product library, demonstrating robust predictive performance and capturing essential pharmacophoric motifs vital for identifying potent MraY inhibitors.

### 3.3 Identification of CNP0387675 through pharmacophore screening and cross-structure docking

**Figure 2:**
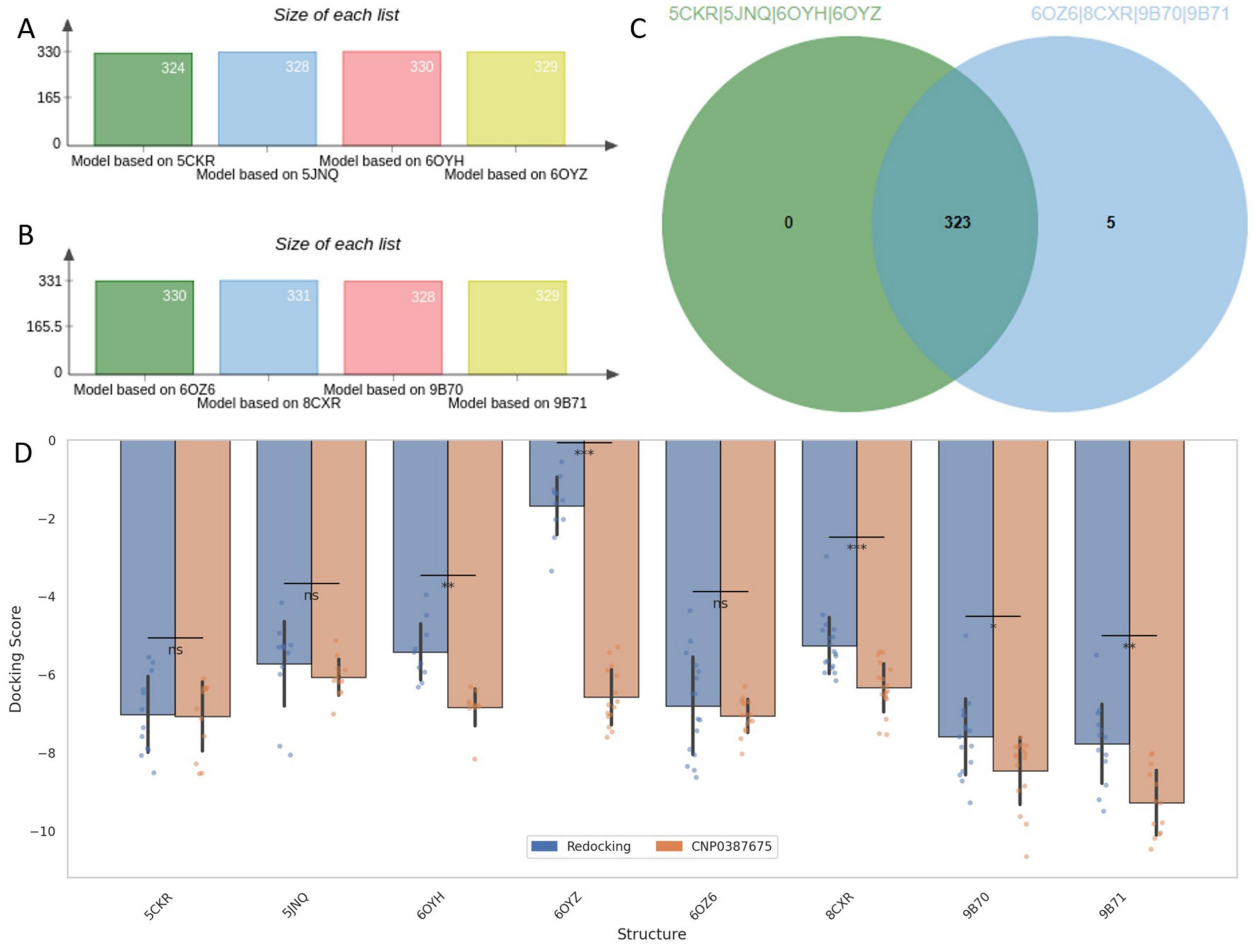
Pharmacophore-based screening and cross-docking evaluation of candidate compounds. **(A–C)** Number of compounds retained by AAAR_3 pharmacophore screening across eight *MraY* crystal structures. **(D)** Comparison of Induced Fit Docking scores between reference ligands and the selected candidate CNP0387675 across all protein models.

Leveraging the validated AAAR_3 pharmacophore hypothesis, we conducted a structure-based virtual screening campaign targeting *MraY* using a comprehensive natural product library. The AAAR_3 model was applied to all eight ligand-bound *MraY* crystal structures to accommodate structural variability across the ensemble. As shown in Figures 2A–C, approximately 330 compounds passed the pharmacophore filtering step for each structure. The compound set consensus across all structures yielded 323 unique candidates for further evaluation.

To assess binding potential, receptor grids were constructed based on the co-crystallized ligand binding sites of each *MraY* structure. All 323 filtered natural products were subjected to Glide docking, and the co-crystallized reference ligands were redocked under identical conditions as performance benchmarks. Among all screened compounds, only one—CNP0387675—consistently achieved docking scores that outperformed the redocked reference ligands across all eight protein structures.

Recognizing the limitations of standard semi-flexible docking, which assigns a single score per ligand and may fail to identify optimal poses in flexible or diverse binding sites, we employed Induced Fit Docking (IFD) to enhance pose reproducibility and sampling depth. This protocol retained up to 20 binding poses per ligand per structure, providing a more complete view of the conformational binding landscape. A summary of the comparative docking scores between redocked ligands and CNP0387675 across all targets is shown in Figure 2D. Paired *t*-tests were performed to evaluate statistical significance. While CNP0387675 exhibited docking scores comparable to the redocked ligands in most structures, significantly improved scores (*p* < 0.05) were observed in several key targets, including 6OYH, 8CKR, 9B70, and 9B71. These results highlight the potential of CNP0387675 to achieve preferential binding at specific conformational states or active site variants of *MraY*.

Given its favorable cross-structure performance, CNP0387675 was selected for subsequent molecular dynamics simulations to assess binding stability and interaction consistency over time.

### 3.4 Interaction profiling of CNP0387675 across key target structures reveals conserved binding determinants

**Figure 3:**
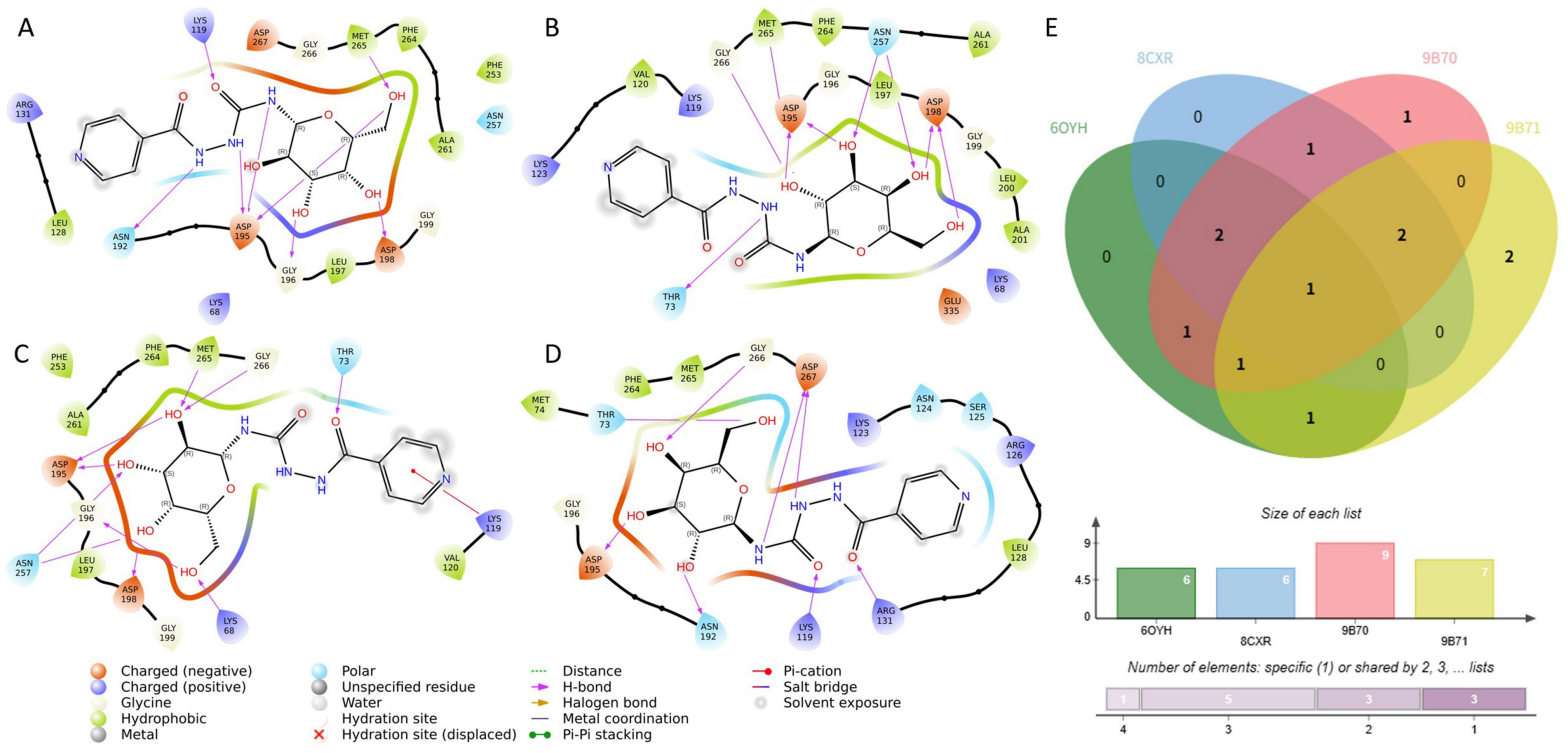
Comparative interaction analysis of CNP0387675 across four *MraY* crystal structures with statistically significant docking results. **(A)** Interactions in the 6OYH complex. **(B)** Interactions in the 8CKR complex. **(C)** Interactions in the 9B70 complex. **(D)** Interactions in the 9B71 complex. **(E)** Summary of conserved and structure-specific interacting residues observed across all four complexes.

To further investigate the binding mechanisms underlying the favorable docking performance of CNP0387675, we conducted detailed interaction analyses with the four *MraY* structures in which statistically significant docking improvements were observed. As illustrated in Figures 3A–D, despite being docked into distinct protein conformations guided by different reference ligand poses, CNP0387675 exhibited a recurring set of interactions that suggest a conserved binding profile. Most notably, all four complexes consistently featured a key interaction with ASP-195, indicating its potential role as a critical anchoring residue.

In addition to this conserved interaction, structure-specific contacts were also observed. Binding to 6OYH, 8CKR, and 9B70 involved additional interactions with ASP-198 and MET-265, while contacts with THR-73 and GLY-266 were common across the 8CKR, 9B70, and 9B71 complexes. The cumulative number of shared and unique interacting residues is summarized in Figure 3E, highlighting the dual nature of CNP0387675’s binding: a conserved core combined with flexible peripheral contacts.

To better understand context-specific binding adaptations, interaction differences across the four complexes were further examined (Supplementary Figure S1). Interestingly, although CNP0387675 often diverged from the original binding mode of the reference ligands, it consistently engaged key functional residues. For example, interactions with LYS-119 and ASN-195 were observed in 6OYH, while binding to 8CKR preserved contact with ASP-195. In 9B70, a broader interaction network involving LYS-68, THR-73, LYS-119, ASP-195, and ASN-257 was observed, whereas the 9B71 complex maintained stable contacts with THR-73, ASP-195, GLY-266, and ASP-267.

Overall, these findings suggest that CNP0387675 may form context-dependent binding modes across different MraY conformations, anchored by a conserved interaction with ASP-195. However, since these poses were obtained through docking and show substantial variation in contact profiles, their functional significance requires further validation. Accordingly, we performed long-timescale molecular dynamics simulations to assess the stability of each pose and identify the most likely biologically relevant conformation.

### 3.5 Conformational stability of CNP0387675 in different crystal structures template of MarY

**Figure 4:**
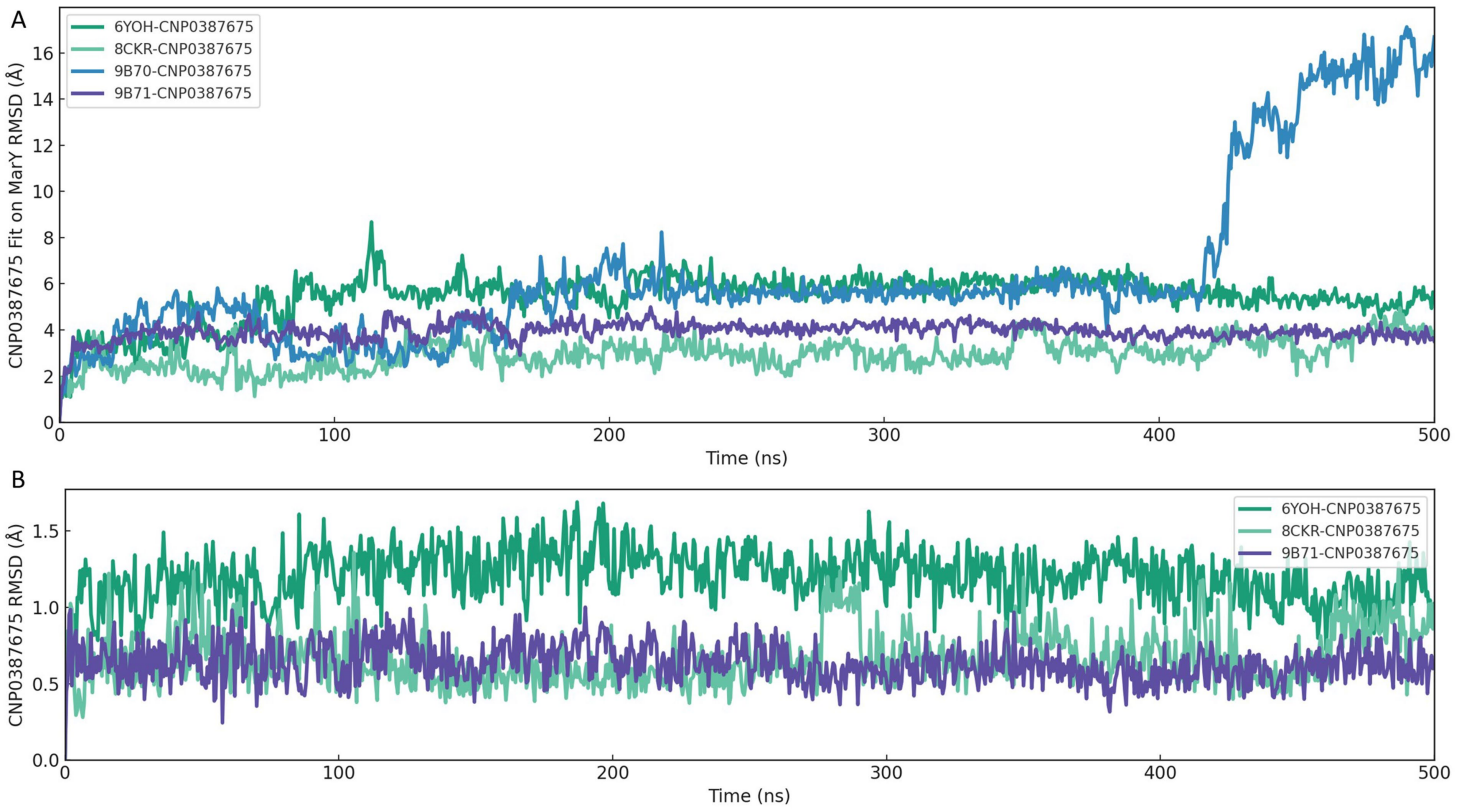
RMSD-based evaluation of CNP0387675 binding stability in four *MraY* crystal structures over 500 ns MD simulations. **(A)** Ligand fit on protein RMSD, reflecting spatial displacement within the binding site. **(B)** Ligand RMSD, indicating internal conformational flexibility.

To assess the binding persistence and conformational stability of CNP0387675 within the active site of MraY, we performed 500 ns classical molecular dynamics (MD) simulations using four selected homology models of *P. aeruginosa* MraY, each derived from a distinct crystal structure template: 6OYH, 8CKR, 9B70, and 9B71. Two RMSD-based metrics were employed: ligand fit on protein RMSD to evaluate spatial displacement within the binding pocket (Figure 4A), and ligand RMSD to assess internal conformational flexibility over time (Figure 4B).

As shown in Figure 4A, the 6OYH system displayed pronounced ligand positional instability, with RMSD values consistently fluctuating between 4 and 6 Å, indicating poor ligand retention within the binding site. Similarly, the 9B70 complex exhibited irregular, non-converging fluctuations ranging from 2 to 5 Å, also indicative of an unstable binding mode. Although the 8CKR complex showed moderate spatial stability (RMSD values ranging from 2.5 to 3.5 Å), its binding mode was not tightly confined, suggesting potential instability over extended simulations. In contrast, the 9B71 system presented the most stable binding profile, characterized by early convergence and sustained RMSD values between 3.5 and 4.2 Å, clearly indicating persistent ligand occupancy within the binding pocket.

Complementary analysis of ligand RMSD (Figure 4B) further supported these findings. The 6OYH ligand exhibited gradual RMSD increases without clear convergence, fluctuating between 1.5 and 2.5 Å, confirming poor internal stability. The ligand in the 8CKR complex displayed somewhat more stable behavior, with values stabilizing between 1.2 and 2.0 Å, though still indicating notable conformational flexibility. Remarkably, the ligand in the 9B71 system rapidly converged within the first 30 ns and maintained consistently low RMSD values (1.0–1.6 Å) throughout the simulation, reflecting minimal internal conformational drift and a stable fit within the binding pocket.

Together, these analyses consistently indicate that only the 9B71-derived pose provides a robust and stable structural environment for CNP0387675. Thus, we identified 9B71 as the sole reliable receptor conformation suitable for further mechanistic investigation and structure-based inhibitor optimization, while other poses were excluded from further detailed analyses due to inadequate stability.

### 3.6 Interaction network analysis reveals key residue engagement in the 9B71–CNP0387675 complex

**Figure 5:**
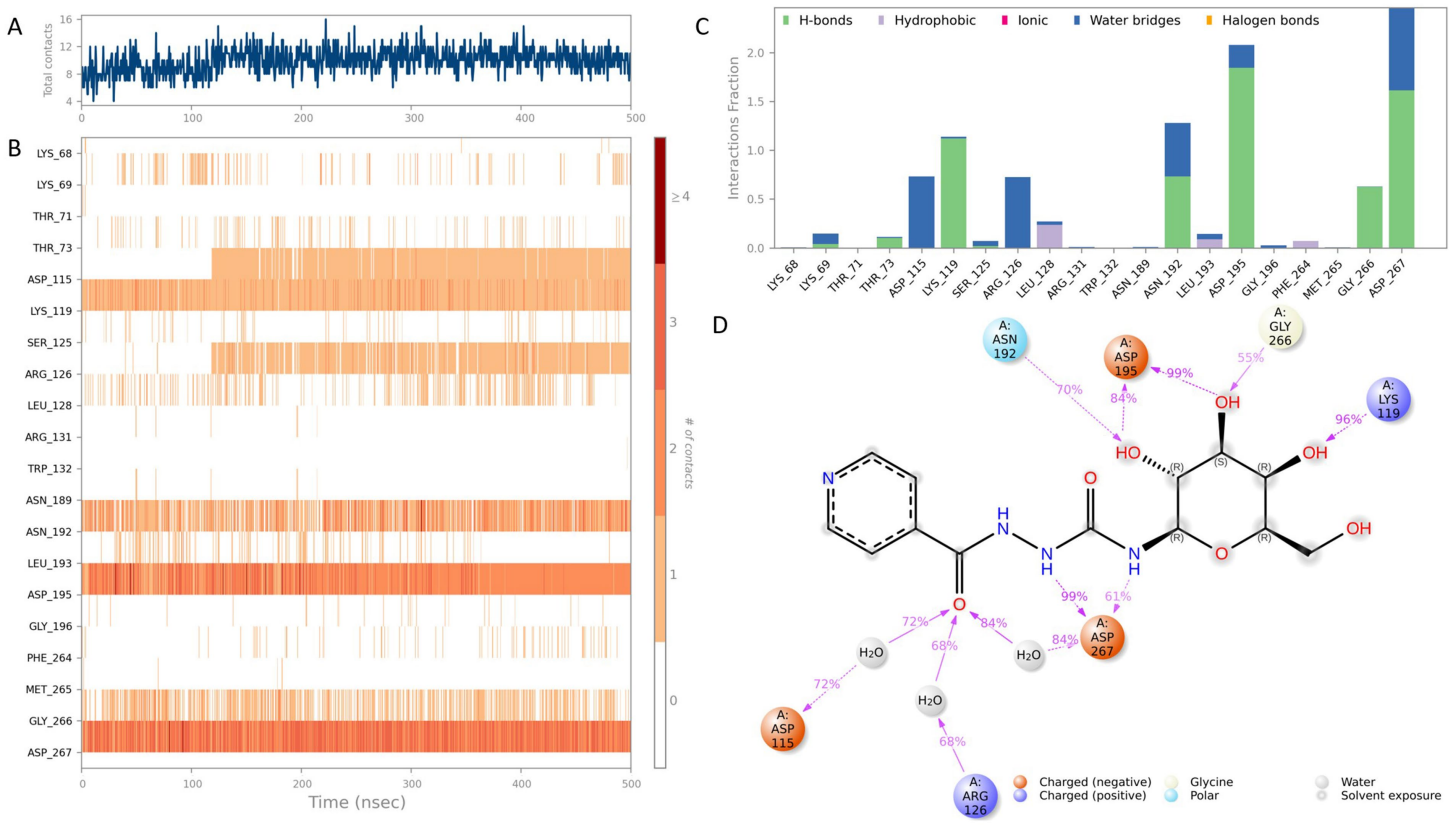
Interaction analysis of the CNP0387675–MraY complex (9B71) over a 500 ns molecular dynamics simulation. **(A)** Number of ligand–protein contacts over time. **(B)** Time-dependent evolution of contact residue identities. **(C)** Classification and frequency of interaction types, including hydrogen bonds, hydrophobic interactions, ionic contacts, water bridges, and halogen bonds. **(D)** Schematic representation of atom-level contacts between CNP0387675 and key residues.

Following confirmation of binding stability through MD simulations, the 9B71–CNP0387675 complex was exclusively selected for detailed interaction dynamics analysis. Throughout the 500 ns molecular dynamics simulation, intermolecular contact patterns were monitored to characterize the evolution of the binding network in detail. As shown in Figures 5A,B, the number of ligand–protein contacts consistently remained around eight during the initial 100 ns and gradually increased to approximately 12 between 100 and 500 ns. Stable and persistent interactions with LYS-119, ASP-195, and ASP-267 were maintained throughout the simulation trajectory. Several additional residues—including ASP-115, ARG-126, ASN-192, and GLY-226—also exhibited frequent contacts (>70% simulation time), suggesting a supportive role in maintaining ligand stability within the binding site.

To further dissect the nature of these interactions, ligand–residue contacts were categorized into five interaction classes: hydrogen bonds, hydrophobic interactions, ionic interactions, water bridges, and halogen bonds. Results summarized in Figures 5C,D highlight multiple high-frequency interaction hotspots, particularly ASP-115, LYS-119, ARG-126, ASN-192, ASP-195, GLY-266, and ASP-267. Among these, ASP-195, LYS-119, and ASP-267 consistently formed direct hydrogen bonds or stable water-mediated bridges with CNP0387675, reinforcing their central role in ligand anchoring. Notably, both ASP-195 and ASP-267 had previously been identified as key residues in the original 9B71 crystal structure, further validating these interactions observed through MD simulations.

### 3.7 Physicochemical and medicinal chemistry evaluation of two MraY inhibitor candidates: CNP0387675 vs. 9B71 Analogue 3

To further contextualize the inhibitory potential of CNP0387675, we conducted a comprehensive comparison with 9B71 Analogue 3, a structurally complex MraY ligand previously validated as an inhibitor. This analysis focused on their respective physicochemical attributes and medicinal chemistry profiles, with the goal of evaluating drug-likeness, developability, and formulation feasibility. The detailed physicochemical parameters are summarized in Table 3, while key medicinal chemistry metrics are provided in Table 4.

**Table 3:** Physicochemical properties of CNP0387675 and 9B71 Analogue 3.

Physicochemical property	CNP0387675	9B71 Analogue 3
Volume	308.628	956.825
Density	1.109	1.005
nHA	11	22
nHD	7	15
nRot	7	35
nRing	2	4
MaxRing	6	6
nHet	11	22
fChar	0	0
nRig	14	28
Flexibility	0.5	1.25
Stereo Centers	5	11
TPSA	173.27	358.92
logS	−0.635	−3.088
logP	−1.756	1.473
logD7.4	−1.158	1.975
pka (acid)	7.072	5.734
pka (base)	3.399	6.597
Melting point	203.465	205.8
Boiling point	345.87	387.785

**Table 4:** Medicinal chemistry and drug-likeness profiles of CNP0387675 and 9B71 Analogue 3.

Medicinal chemistry	CNP0387675	9B71 Analogue 3
QED	0.282	0.026
SAscore	Easy	Easy
GASA	Easy	Hard
Fsp3	0.462	0.696
MCE-18	49.368	102.564
NPscore	−0.15	0.553
Lipinski Rule	Rejected	Rejected
Pfizer Rule	Accepted	Accepted
GSK Rule	Accepted	Rejected
GoldenTriangle	Accepted	Rejected
PAINS	0	0
Alarm_NMR Rule	0	0
BMS Rule	0	1
Chelating Rule	0	0
Colloidal aggregators	0.427	0.591
FLuc inhibitors	0.002	0.001
Blue fluorescence	0.109	0.003
Green fluorescence	0.103	0.232
Reactive compounds	0.01	0.001
Promiscuous compounds	0.002	0.187

This table summarizes core physicochemical descriptors including molecular volume, rotatable bonds, stereocenters, hydrogen bond donors and acceptors, topological polar surface area (TPSA), logP, and aqueous solubility (logS), providing insight into compound size, polarity, and permeability potential.

This table includes drug-likeness scores (QED), synthetic accessibility (SAscore and GASA), molecular complexity indicators (Fsp^3^, MCE-18), natural product-likeness (NPscore), rule-based filters (Pfizer, GSK, Golden Triangle, BMS), and assay interference metrics (PAINS, aggregation score, promiscuity index, fluorescence interference), highlighting their respective developability profiles.

CNP0387675 and 9B71 Analogue 3 exhibit profound differences in size, polarity, and molecular complexity. CNP0387675 is notably smaller in volume (308.63 Å^3^ vs. 956.83 Å^3^), with fewer rotatable bonds (7 vs. 35) and stereocenters (5 vs. 11), indicating a more compact and synthetically tractable structure. These features are favorable for both membrane permeability and manufacturing feasibility. In contrast, the high conformational flexibility and stereochemical richness of 9B71 Analogue 3 could impede its metabolic stability and scale-up potential.

The comparison of polarity and solubility further distinguishes the two compounds. 9B71 Analogue 3 presents an extremely high topological polar surface area (TPSA: 358.92 Å^2^), well above the threshold associated with good membrane permeability and oral bioavailability, whereas CNP0387675 has a TPSA of 173.27 Å^2^—elevated but still within an optimizable range. Moreover, while 9B71 Analogue 3 displays higher lipophilicity (logP = 1.47), its aqueous solubility is markedly poor (logS = −3.09), in contrast to CNP0387675’s more soluble profile (logS = −0.64), albeit at the expense of lower passive permeability (logP = −1.76).

From a medicinal chemistry perspective, CNP0387675 shows a more balanced and favorable profile. It achieves a quantitative estimate of drug-likeness (QED) score of 0.282, significantly outperforming 9B71 Analogue 3 (QED = 0.026). Synthetic accessibility, as evaluated by graph-based and substructure-based algorithms, further supports this distinction—although both compounds were rated “easy” in fragment-based SAscore, the GASA score classifies 9B71 Analogue 3 as synthetically “hard.” Notably, 9B71 Analogue 3 also violates multiple lead-likeness criteria, including the GSK and Golden Triangle filters, suggesting potential liabilities in its absorption, distribution, metabolism, and excretion (ADME) properties.

In terms of screening compatibility, neither compound triggers PAINS or chelator alerts. However, 9B71 Analogue 3 shows a higher colloidal aggregation score (0.591 vs. 0.427), elevated fluorescence interference (notably in the green spectrum), and a substantially higher promiscuity score (0.187 vs. 0.002 for CNP0387675), indicating a greater risk of non-specific activity and false positives in biochemical assays.

Taken together, this comparative analysis underscores the superior developability of CNP0387675. While it may require optimization to improve membrane permeability, its overall drug-likeness, solubility, structural simplicity, and interaction specificity render it a more promising lead compound for further preclinical development. In contrast, 9B71 Analogue 3, despite its previously validated bioactivity, presents multiple chemical liabilities that would likely necessitate substantial structural simplification and optimization before advancing as a drug candidate.

### 3.8 Comparative analysis of binding stability between CNP0387675 and 9B71 Analogue 3

**Figure 6:**
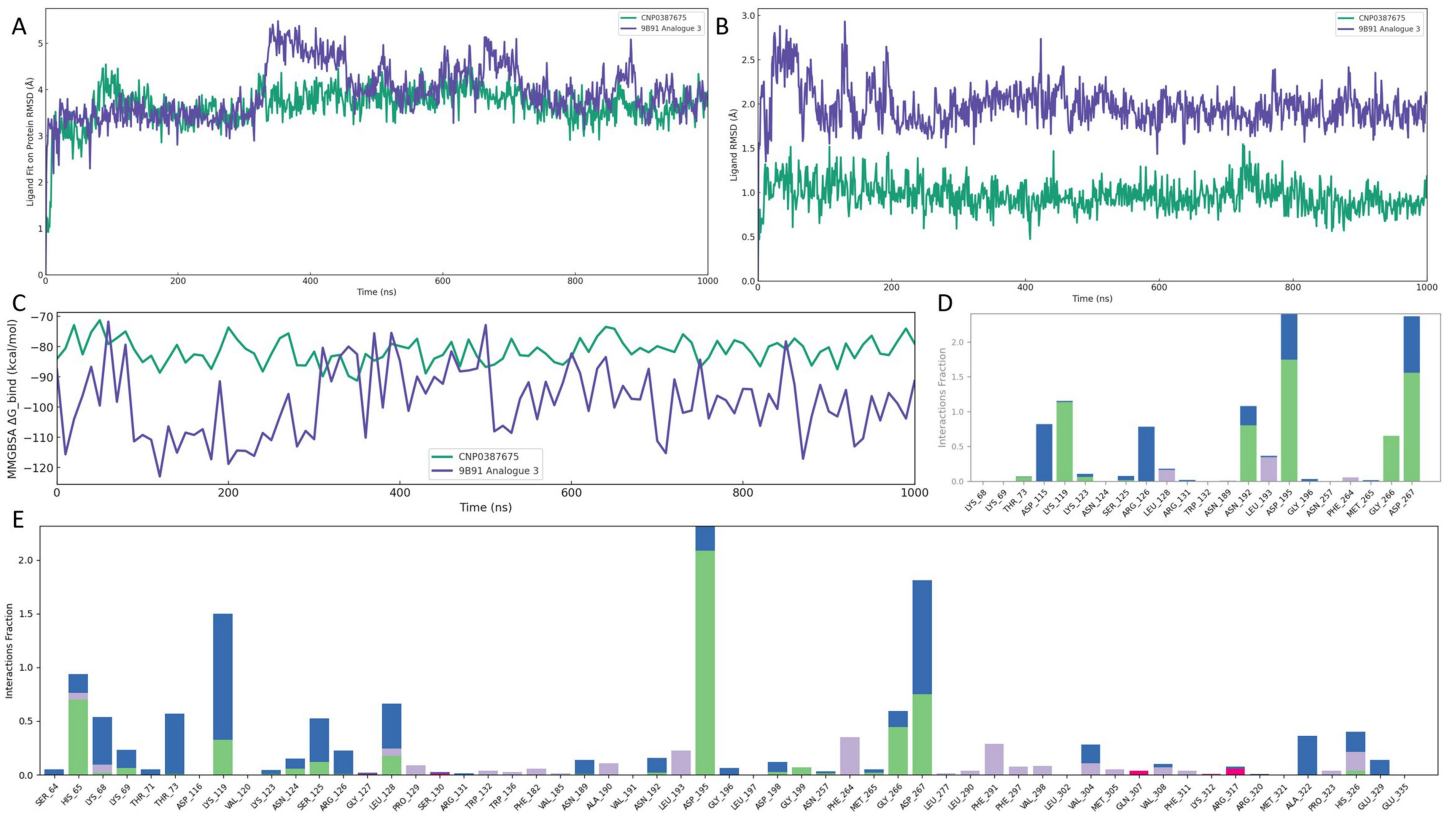
Comparative binding stability and interaction profiling of CNP0387675 and 9B71 Analogue 3 in the *MraY* binding site over 1,000 ns MD simulations. **(A)** Ligand fit on protein RMSD. **(B)** Ligand internal RMSD. **(C)** MMGBSA binding free energy (ΔG_bind) at 10 ns intervals. **(D)** Per-residue interaction frequency of CNP0387675. **(E)** Per-residue interaction frequency of 9B71 Analogue 3.

To comprehensively assess the binding stability and performance of CNP0387675 relative to 9B71 Analogue 3, we conducted 1,000 ns molecular dynamics simulations and analyzed multiple complementary descriptors: ligand fit on protein RMSD (Figure 6A), ligand RMSD (Figure 6B), MMGBSA binding free energy (Figure 6C), and interaction frequency distributions (Figures 6D,E). These parameters collectively capture spatial retention, conformational rigidity, thermodynamic favorability, and interaction persistence within the *MraY* binding pocket.

As shown in Figure 6A, CNP0387675 exhibited a consistently lower and more stable ligand fit on protein RMSD trajectory than 9B71 Analogue 3. Following an initial adjustment period, CNP0387675 stabilized within a narrow range of 1.0–2.5 Å, whereas 9B71 Analogue 3 frequently exceeded 3.0 Å without achieving convergence throughout the simulation. Likewise, Figure 6B indicated that CNP0387675 maintained minimal internal deviation (0.5–1.5 Å), in contrast to the larger and more erratic fluctuations (1.5–3.0 Å) observed for 9B71 Analogue 3.

To evaluate binding thermodynamics, MMGBSA binding free energy (ΔG_bind) was calculated at 10 ns intervals (Figure 6C). CNP0387675 maintained stable and favorable free energy values ranging from −70 to −90 kcal/mol, with minimal variation. Although 9B71 Analogue 3 occasionally reached more negative ΔG_bind values (dropping below −110 kcal/mol), these events were accompanied by high volatility and lacked temporal consistency. It is worth noting that the stronger energetic contributions observed for 9B71 Analogue 3 may partially result from its larger molecular size and greater number of heavy atoms, which inherently promote more extensive van der Waals and electrostatic interactions. However, this apparent advantage is undermined by its diffuse binding mode and poor structural convergence.

Protein–ligand interaction analysis further revealed qualitative differences in binding specificity (Figures 6D,E). Under physiological simulation conditions, CNP0387675 consistently engaged a focused subset of high-frequency residues, including ASP-115, LYS-119, ARG-126, ASN-192, LEU-193, ASP-195, GLY-266, and ASP-267. These contacts were also observed in prior simulation systems, suggesting a reproducible and coherent binding mode. In contrast, 9B71 Analogue 3 exhibited a broader but less organized interaction profile, engaging a greater number of residues with sporadic and less persistent contacts.

Taken together, these results demonstrate that CNP0387675 exhibits superior structural stability, interaction specificity, and thermodynamic reliability compared to 9B71 Analogue 3. Its consistent pose retention, focused and reproducible contact network, and stable binding free energy profile support its candidacy as a structurally robust and pharmacologically viable *MraY* inhibitor under simulated physiological conditions.

### 3.9 Comprehensive analysis of structural compactness and surface exposure in ligand–*MraY* complexes

**Figure 7:**
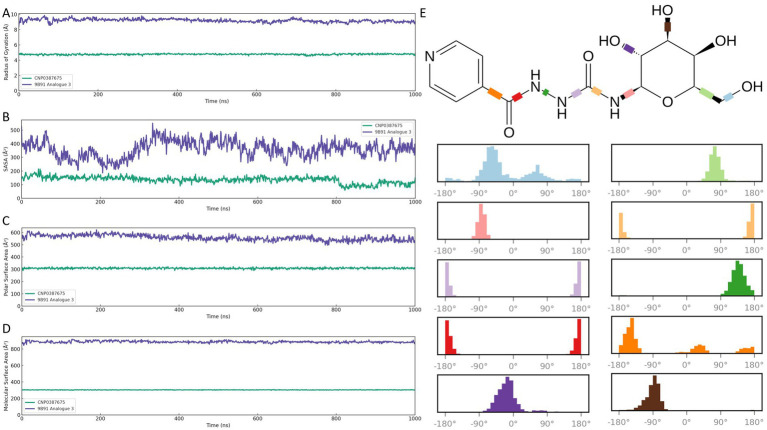
Global structural and surface property analyses of CNP0387675 and 9B71 Analogue 3 in complex with *MraY* over 1,000 ns molecular dynamics simulations. **(A)** Radius of gyration (Rg). **(B)** Solvent-accessible surface area (SASA). **(C)** Polar surface area (PSA). **(D)** Molecular surface area (MSA). **(E)** Distribution of torsional angles of the ten rotatable bonds in CNP0387675, highlighting its concentrated conformational sampling and enhanced atomic-level stability compared with the broader torsional flexibility of 9B71 Analogue 3.

To further characterize the global structural stability and surface properties of the ligand–*MraY* complexes, we evaluated four key descriptors over 1,000 ns classical molecular dynamics simulations: radius of gyration (Rg), solvent-accessible surface area (SASA), polar surface area (PSA), and molecular surface area (MSA) (Figures 7A–D). Together, these parameters reflect the extent of structural compactness, solvent exposure, and spatial definition of the ligand–protein assemblies under dynamic conditions.

As shown in Figure 7A, CNP0387675 maintained a compact complex architecture throughout the trajectory, with Rg values fluctuating narrowly between ~4.7 and 4.9 Å. This suggests a tightly packed and conformationally restrained binding mode. In contrast, 9B71 Analogue 3 exhibited substantially higher Rg values (~8.3–9.5 Å) with broader fluctuations, indicative of a more extended and flexible conformation.

Surface exposure analyses corroborated these differences. In Figure 7B, CNP0387675 showed consistently lower SASA values (~140–160 Å^2^), suggesting limited solvent interaction and a more buried binding profile. Conversely, 9B71 Analogue 3 displayed markedly elevated SASA values (~370–530 Å^2^), reflecting greater exposure of the ligand–protein interface to the aqueous environment.

Further decomposition of surface polarity (Figure 7C) revealed that CNP0387675 maintained stable PSA values (~290–320 Å^2^), indicating a moderately polar but shielded interface. In comparison, 9B71 Analogue 3 exhibited higher and more variable PSA values (~540–580 Å^2^), consistent with increased solvent interaction and greater conformational flexibility.

Finally, molecular surface analysis (Figure 7D) reinforced these observations. CNP0387675 retained a well-defined and geometrically stable MSA (~300–310 Å^2^), while 9B71 Analogue 3 presented a significantly larger and irregular MSA (~860–880 Å^2^), suggestive of a less constrained and dynamically fluctuating structure. As shown in Figure 7E, CNP0387675 contains ten rotatable bonds, and their torsional angles remained relatively concentrated throughout the simulation, indicating a higher degree of atomic-level stability. Collectively, these compactness and surface-based evaluations support a consistent conclusion: CNP0387675 forms a more stable, compact, and spatially confined complex with MraY than 9B71 Analogue 3. These findings align with prior RMSD, binding free energy, and interaction network results, reinforcing the superior structural integrity and binding efficiency of CNP0387675 under physiologically simulated conditions.

Collectively, these compactness and surface-based evaluations support a consistent conclusion: CNP0387675 forms a more stable, compact, and spatially confined complex with *MraY* than 9B71 Analogue 3. These findings align with prior RMSD, binding free energy, and interaction network results, reinforcing the superior structural integrity and binding efficiency of CNP0387675 under physiologically simulated conditions.

### 3.10 Ligand-induced conformational locking and dynamic suppression in *MraY*

**Figure 8:**
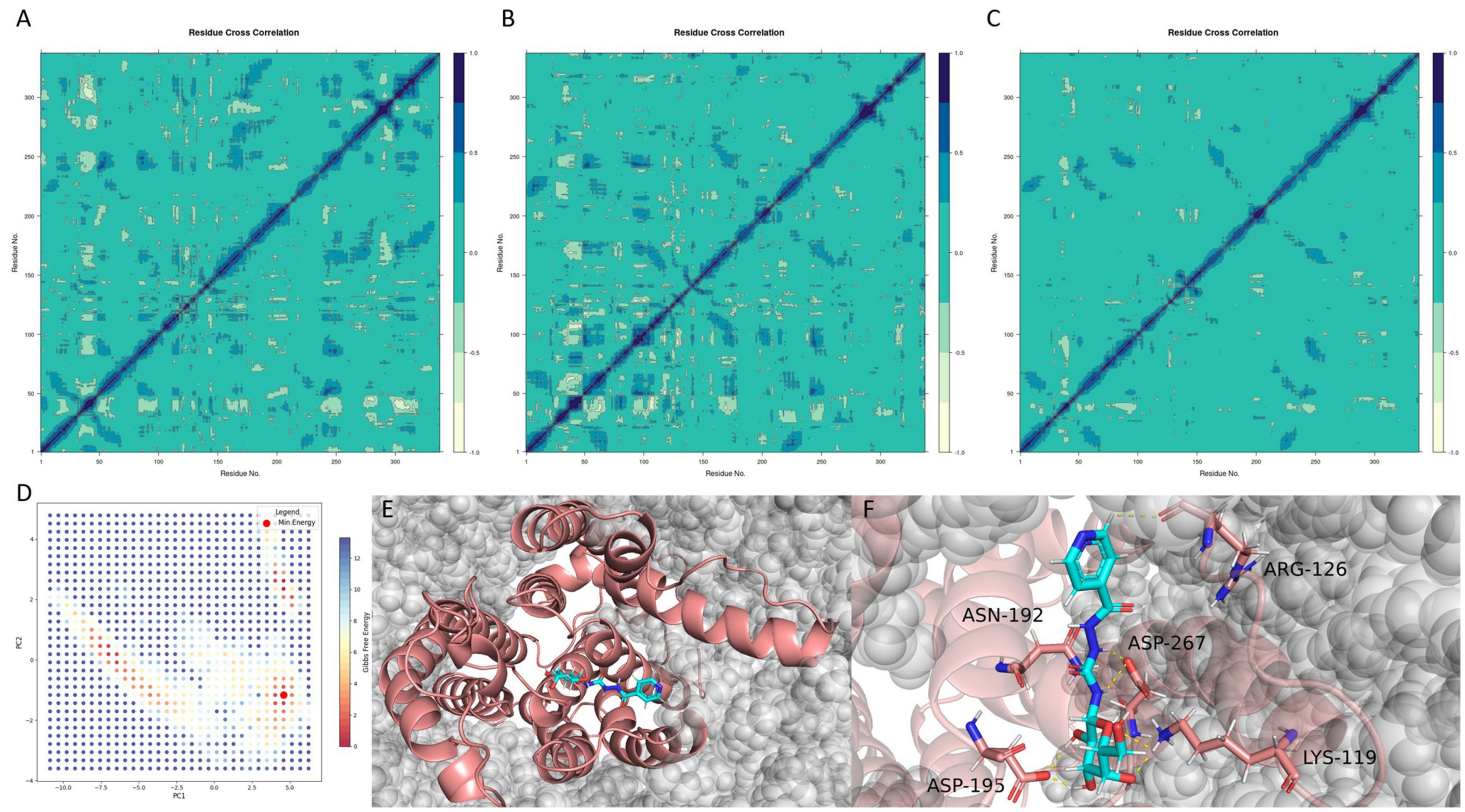
Ligand-induced dynamic modulation and conformational stabilization of *MraY*. **(A–C)** Dynamic cross-correlation matrices (DCCMs) of the apo protein, 9B71 Analogue 3–bound complex, and CNP0387675–bound complex, respectively. **(D)** Free energy landscape (FEL) of the CNP0387675 complex derived from the first two principal components (PC1 and PC2). **(E)** Global minimum conformation extracted from the FEL surface. **(F)** Representative interaction network in the minimum-energy binding pose.

To investigate how ligand binding modulates the internal dynamics and conformational preferences of *MraY*, we conducted a comprehensive analysis of protein motion in the apo form and two ligand-bound complexes (CNP0387675 and 9B71 Analogue 3). This included dynamic cross-correlation matrices (DCCMs), principal component analysis (PCA), and free energy landscape (FEL) mapping (Figure 8).

As shown in Figure 8A, the apo *MraY* protein exhibited widespread long-range positive correlations across distal residue pairs, reflecting a highly flexible and communicative internal dynamic network. Upon binding of 9B71 Analogue 3 (Figure 8B), these correlations were markedly reduced and became more localized along the diagonal of the DCCM, indicating a shift toward localized motions and partial conformational rigidification.

This dampening effect was even more pronounced in the CNP0387675-bound complex (Figure 8C). Compared to the apo state, the DCCM of the CNP0387675 system revealed an extensive loss of dynamic coupling, characterized by weak, fragmented correlations across most residue pairs. This suggests that CNP0387675 imposes a more substantial conformational constraint, effectively locking the protein into a less dynamically permissive state.

Together, these results indicate that both ligands suppress MraY’s intrinsic flexibility, consistent with inhibitory binding at or near functionally important regions. The greater suppression observed with CNP0387675 may reflect a tighter and more persistent engagement with the catalytic core, contributing to enhanced structural stabilization.

To further identify the predominant conformational states stabilized by CNP0387675, we performed PCA and FEL analyses based on the first two principal components (PC1 and PC2) (Figure 8D). The resulting free energy surface revealed a single dominant energy basin, suggesting that the protein–ligand system converges into a well-defined, thermodynamically favorable conformation. This global minimum was extracted and structurally characterized (Figures 8E,F).

Interestingly, comparison with previous interaction frequency analyses revealed that although ASP-115 and GLY-266 frequently interacted with CNP0387675 during the simulation, they were not involved in the final minimum-energy pose. This finding highlights the transient nature of early binding intermediates and suggests that only a subset of interactions persists in the most stable binding mode. In contrast, residues such as LYS-119, ASP-195, and ASP-267 consistently formed stabilizing contacts in both dynamic and energy-minimized configurations.

Taken together, these findings demonstrate that CNP0387675 not only suppresses global dynamic fluctuations more effectively than 9B71 Analogue 3, but also stabilizes *MraY* in a distinct low-energy conformation. The convergence of dynamic dampening and FEL-defined structural rigidity underscores its potential as a conformational inhibitor, offering mechanistic insights into its enhanced binding stability and therapeutic promise.

### 3.11 Binding pose robustness and residue importance validated by alanine scanning and pose metadynamics

**Figure 9:**
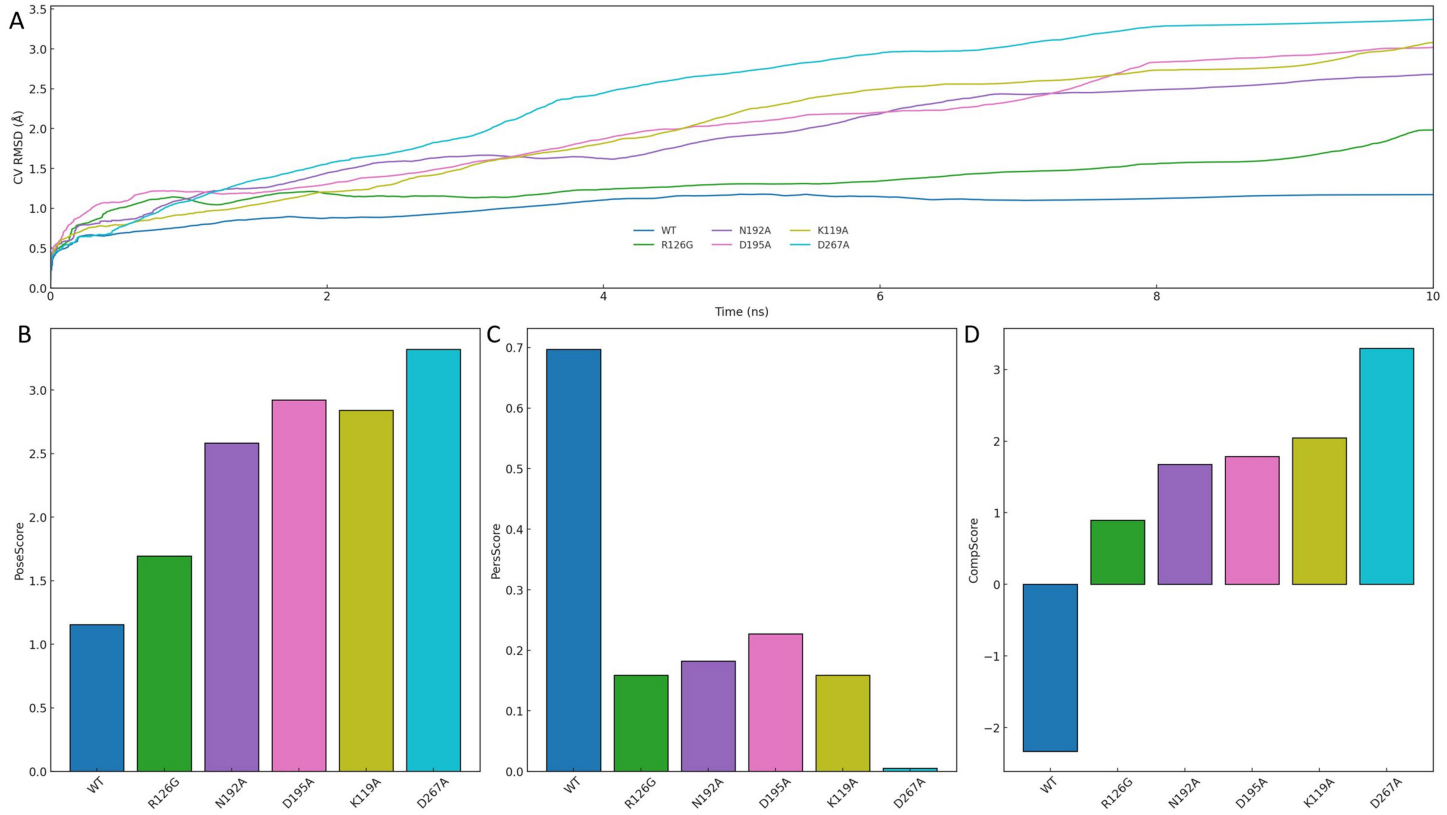
Binding pose stability assessment through alanine scanning and metadynamics simulation. **(A)** CV RMSD trajectories comparing the wild-type (WT) and alanine-substituted mutant systems over 10 ns. **(B)** PoseScore indicating deviation from initial binding conformation. **(C)** PersScore reflecting interaction persistence with the protein pocket. **(D)** CompScore representing the overall quality of the binding pose.

To assess the structural relevance of the interaction pattern derived from the minimum free energy conformation, each key residue was subjected to alanine substitution, and the resulting systems were evaluated through binding pose metadynamics simulations. As shown in Figure 9A, the CV RMSD of the wild-type (WT) system remained consistently low throughout the 10 ns simulation, reflecting a highly stable binding pose. In contrast, all mutants exhibited elevated CV RMSD values, indicating greater conformational fluctuation and reduced pose retention upon disruption of individual interactions.

Quantitative scoring results further support this observation. As presented in Figures 9B–D, WT achieved the most favorable profile across all metrics: the lowest PoseScore, indicative of minimal deviation from the initial conformation; the highest PersScore, reflecting persistent interaction with the protein pocket; and the highest CompScore, demonstrating overall superiority in binding pose quality. In comparison, mutations at residues such as K119A, D195A, and D267A led to pronounced declines across all three scores, underscoring the structural significance of each site.

Taken together, these results confirm that each residue involved in the minimum-energy conformation is essential for stabilizing ligand binding. The consistent loss of pose integrity and interaction persistence upon single-point mutation highlights the importance of maintaining a complete interaction network. Therefore, the interaction pattern observed in the FEL global minimum is not only energetically optimal but also functionally indispensable, providing structural validation of its role in mediating high-affinity ligand recognition.

### 3.12 Antimicrobial activity validation of CNP0387675 against *P. aeruginosa* PAO1 (ATCC 15692, 1C, PRS 101) and *E. bolteae* 90A9

Strain selection was anchored to the structural templates used in our MraY work and to the feasibility of standardized testing at 37 °C with OD₆₀₀ readouts. The in-silico component relied on MraY structures from Aquifex aeolicus VF5 (PDB 5CKR) and Enterocloster (Clostridium) bolteae 90A9 (PDB 5JNQ). Because *A. aeolicus* is a hyperthermophile with growth optima around or above 80–90 °C, the conventional 37 °C broth-microdilution workflow is not applicable in our laboratory setting; consequently, it was referenced structurally but excluded from phenotypic testing. By contrast, E. bolteae 90A9 is routinely cultivated at 37 °C under strict anaerobiosis in DSMZ-recommended media and was therefore selected as the additional validation species. For the aerobic arm, we focused on *Pseudomonas aeruginosa* PAO1 lineage, cataloged under multiple accessions (ATCC 15692/DSM 22644/CIP 104116/JCM 14847/LMG 12228/1C/PRS 101/PAO1), and chose three widely used derivatives that are conventionally grown at 37 °C—ATCC 15692 (PAO1), 1C, and PRS 101—to harmonize phenotypic assays with the structural inputs.

**Figure 10:**
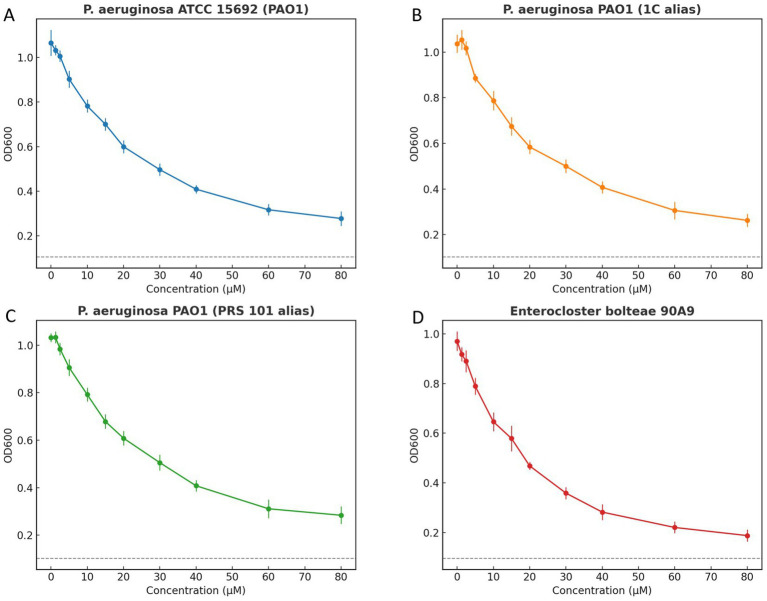
Growth inhibition curves of *Pseudomonas aeruginosa* and *Enterocloster bolteae* exposed to increasing compound concentrations. Panels show **(A)** *P. aeruginosa* ATCC 15692 (PAO1), **(B)** *P. aeruginosa* PAO1 (1C alias), **(C)** *P. aeruginosa* PAO1 (PRS 101 alias), and **(D)** *E. bolteae* 90A9. Points indicate mean ± SD of OD600 at 18 h from three independent replicates. A dashed horizontal line represents an operational “no-growth” threshold at 10% of the control mean OD, included for visualization only. Internal gridlines were removed, and each panel uses a distinct color scheme with border-only top x-axis and right y-axis. Minimum inhibitory concentration (MIC) determination in the text follows CLSI visible-growth criteria.

Using CNP0387675 across 0–80 μM, all four strains exhibited a monotonic decline in OD₆₀₀ with increasing concentration, with the principal inflection typically between ~15 and 30 μM. Residual growth persisted at 80 μM; therefore, following the CLSI visible-growth criterion, the MIC for each strain in this experiment is reported as ≥ 80 μM. The OD-based curves and the overlaid 10%-of-control “no-growth” threshold, provided solely for visualization, are concordant with the MIC calls by showing incomplete suppression within the assayed range. Under strict anaerobic conditions, E. bolteae 90A9 produced a response pattern consistent with the aerobic PAO1 panels, indicating comparable concentration dependence when DSMZ-recommended media and anaerobic handling were applied. Replicate dispersion was modest and trajectories were smooth, supporting technical reproducibility while emphasizing that formal susceptibility reporting relies on the CLSI MIC endpoint defined by absence of visible growth (Figure 10).

## 4 Discussion

Our study presents an integrated computational pipeline aimed at systematically identifying novel inhibitors of MraY, a critical bacterial enzyme involved in peptidoglycan biosynthesis. By employing a structure-based modeling strategy using multiple single-template homology models, pharmacophore-guided virtual screening, multi-template docking, molecular dynamics (MD) simulations, and computational evaluation of drug-likeness and medicinal chemistry-related properties, we addressed the structural and mechanistic challenges associated with targeting MraY.

A key advantage of this computational approach is the utilization of multi-template homology modeling (Larsson et al., 2008; Koehler Leman and Bonneau, 2023). Due to the lack of experimentally determined high-resolution structures for *P. aeruginosa* MraY, we chose instead to use homology models. The homology models were constructed using multiple ligand-bound templates derived from various bacterial species, since that is better than building the models based on only one template (Qian et al., 2007). This approach effectively captures conformational heterogeneity and flexibility inherent in integral membrane proteins, thus enabling a more representative binding-site landscape for subsequent ligand discovery (Varadi et al., 2023). Multi-template docking across these validated receptor models enhanced the accuracy of ligand binding predictions by mitigating biases from single-template docking and ensuring robust candidate selection (Mohammadi et al., 2022; McKay et al., 2022). Furthermore, pharmacophore-based virtual screening, guided by conserved interaction features identified from known inhibitors, effectively enriched the candidate pool, rapidly identifying the novel non-nucleoside inhibitor, CNP0387675 (Chen et al., 2009). Notably, CNP0387675 exhibited consistently superior docking scores across multiple receptor conformations, underscoring its potential as a bona fide inhibitor candidate (Totrov and Abagyan, 2008; Palacio-Rodríguez et al., 2019).

Subsequent MD simulations provided crucial validation of binding stability and mechanistic action (Pandya et al., 2024). Simulations revealed stable interactions between CNP0387675 and conserved catalytic residues (ASP-195 and ASP-267), suggesting a robust allosteric mechanism characterized by induced conformational rigidity in MraY. Compared to established nucleoside analog inhibitors, such as the 9B71 Analogue 3, CNP0387675 demonstrated enhanced suppression of dynamic protein fluctuations, indicative of its potential inhibitory efficacy.

Early medicinal chemistry evaluations further strengthened the candidate’s profile. Compared to the co-crystallized nucleoside inhibitor 9B71 Analogue 3, CNP0387675 exhibited superior drug-like attributes. Its favorable physicochemical characteristics—including reduced molecular complexity, fewer rotatable bonds, decreased stereochemical diversity (Luo et al., 2019), and low aggregation propensity (Navarro and Ventura, 2022)—markedly enhance its feasibility for preclinical drug development.

We extend our computational insights with preliminary antimicrobial testing of CNP0387675 against *P. aeruginosa* PAO1 lineage strains (ATCC 15692, 1C, PRS 101) and the strict anaerobe Enterocloster (Clostridium) bolteae 90A9 under standardized 37 °C broth microdilution conditions. All four strains exhibited a concentration-dependent suppression of OD₆₀₀ with the principal inflection occurring between ~15–30 μM, yet residual growth persisted at the maximum tested concentration (80 μM), thus the MIC was determined to be ≥ 80 μM per CLSI visible-growth criteria. The OD-based dose–response curves and the illustrative 10%-of-control “no-growth” threshold align with these MIC determinations. Under anaerobic conditions, E. bolteae 90A9 showed a response pattern congruent with the PAO1 strains, confirming assay robustness across microbial types. Replicate variability was low and the trajectories smooth, emphasizing technical reproducibility, while formal susceptibility reporting remains tied to the CLSI-defined visible-growth endpoint.

While these results signal tangible *in vitro* activity, CNP0387675 remains several orders of magnitude less potent than clinically relevant antibiotics—highlighting the need for further scaffold optimization to lower effective concentrations toward physiologically viable ranges. Future efforts will prioritize structure–activity relationship (SAR) campaigns derived from docking, MD simulations, and emerging mechanistic insights such as those reported by Yamamoto et al. in their successful optimization of MraY inhibitor analogs showing activity against drug-resistant strains in vitro and *in vivo*. These medicinal chemistry initiatives will be supported by biophysical validation (e.g., crystallography, cryo-EM, SPR, ITC) of compound–MraY interactions. Multidisciplinary collaborations integrating microbiology, structural biology, and computational modeling will be essential to accelerate transition from computational lead to preclinical candidate.

In conclusion, the integration of computational modeling with preliminary antimicrobial validation identifies CNP0387675 as a viable scaffold for MraY inhibition, yet underscores the critical developmental gap that remains before clinical applicability can be achieved. This work provides both mechanistic foundation and strategic direction for downstream inhibitor optimization and antibiotic innovation.

## 5 Conclusion

This integrated study combines sophisticated computational modeling—including pharmacophore-guided multi-template homology modeling, docking, and molecular dynamics validation—with preliminary in vitro validation to characterize CNP0387675 as a promising non-nucleoside MraY inhibitor. Broth microdilution assays against *P. aeruginosa* PAO1 derivatives (ATCC 15692, 1C, PRS 101) and E. bolteae 90A9 demonstrated concentration-dependent inhibition, with MIC values of ≥ 80 μM under CLSI^®^ visible-growth criteria. While this confirms the compound’s biological activity, the modest potency underscores the gap between current in vitro results and therapeutic applicability. Guided by mechanistic insights—particularly those elucidated in Yamamoto et al.’s recent MraY inhibitor optimization—future efforts will prioritize structure–activity relationship-driven scaffold refinement to lower effective concentrations and enhance pharmacokinetic properties. Such optimization, combined with biophysical verification and multi-disciplinary collaboration, will be essential for advancing CNP0387675 toward a viable therapeutic lead against antimicrobial-resistant pathogens.

## Data Availability

The raw data supporting the conclusions of this article will be made available by the authors, without undue reservation.

## Footnotes

^1^ https://www.uniprot.org

^2^ https://www.rcsb.org/

^3^ https://admetlab3.scbdd.com/
